# Supplementary material for: Divergence in alternative polyadenylation contributes to gene regulatory differences between humans and chimpanzees
Source: eLife. 2021 Feb 17;10:e62548. doi: 10.7554/eLife.62548 (PMC7954529; doi:10.7554/eLife.62548)
Supplement: Peer review history

# Decision letter

**DOI:** 10.7554/eLife.62548.sa1

**Affiliations:** University of California, DavisUnited States

In the interests of transparency, eLife publishes the most substantive revision requests and the accompanying author responses.

Thank you for submitting your article "Divergence in alternative polyadenylation contributes to gene regulatory differences between humans and chimpanzees" for consideration by *eLife*. Your article has been reviewed by three peer reviewers, and the evaluation has been overseen by a Reviewing Editor and Naama Barkai as the Senior Editor. The reviewers have opted to remain anonymous.

The reviewers and Reviewing Editor have discussed the reviews with one another and drafted this decision to help you prepare a revised submission. The reviewers and I all agree that the paper is suitable for publication in *eLife*. From our discussion we agree that no additional experiments are necessary but that some additional analyses would help flesh out the biological conclusions of the paper. The reviewers all read over each other’s comments and thought the requested analyses seemed reasonable. I have included the full reviews below, for you to provide a point-by-point response to.

Reviewer #1:

The paper is a well-executed and thorough analysis of PAS usage in LCLs between humans and chimpanzees. The research is technically sound, and this is additionally demonstrated by the accompanying supplemental analyses, and the use of additional datasets which permitted studying how differential PAS usage relates to protein level expression. Yet the paper lacks direct connections to actual phenotypic and biological differences between humans and chimpanzees, and in this regard reads more of a technical work (perhaps suitable in its current form in a journal such as Genome Research or Nucleic Acids Research), than the type of study that is published on a specific biological phenotype as often presented in *eLife*. Along with additional issues listed below, are suggestions for adjusting the analyses and text to help bridge the gap to phenotype:

1) Throughout the Results sections the authors present a myriad of lists of gene and PAS usage sites that result from different ways of cutting the data and connecting PAS usage to isoform and protein expression. Can these lists be explored in more detail, perhaps through functional gene set enrichment analyses and/or the use of the GREAT? Analyzing the sets in at least this manner might help to connect PAS usage differences to actual biology between humans/chimpanzees as well as within each species.

2) While this reviewer greatly appreciated the assessment of PAS via 3' Seq along in tandem with mRNA expression, and in the context of their incorporation of published protein and isoform data on the same cell type, another main issue is the lack of any functional validation experiments for any of the insights they generate in each of the Results sections. This is a bit concerning considering the reliance on LCLs as a standalone cell type for this work.

Reviewer #2:

Mittleman and colleagues have used 3' RNA-seq to study change in polyadenylation site usage between humans and chimpanzees. The results I suspect will be of greatest interest are that PAS change is associated with change in both RNA and protein level gene expression. This was a very interesting study, and something I hadn't considered before. The data collected seems to be a perfect fit for their study and the general analysis approach is solid.

1) I had one major concern with the manuscript as it's written-throughout the results, effect sizes were consistently weak to moderate at best (1-1.5X enrichment, weak correlations, etc.). This in itself is fine. I strongly believe there is often too much emphasis on results with large effect sizes. That said, the Discussion makes some strong claims about their data without tempering their language to account for the effect size. Some additional background information contrasting their results with other aspects of the transcription process would likely help place the role of PAS change in gene expression divergence between species. For example, how strongly does change in ChIP-seq/ATAC-seq/DNase-seq signal associate with DE genes between species. Basically, statements like "We showed that, across species, increased intronic PAS usage is associated with increased mRNA expression levels, while increased 3' UTR PAS usage is correlated with a decrease in mRNA expression" could use some additional context to highlight the relative significance of this result.

2) "Indeed, we found that inter-species differences in the usage of intronic and 3' UTR PAS correlate with differences in expression effect size between the species at an equal magnitude, but in opposite directions (Figure 3B). Increased usage of intronic sites is correlated with increased expression levels, while increased usage of 3' UTR sites is correlated with decreased expression."

It's unclear why the uncategorized correlation (-0.06) was deemed "not meaningful", yet the categorized correlations (-0.077, 0.073) were considered to be "correlated". Neither of these seem particularly well correlated to me.

3) Figure 6 could use additional null models to determine whether these findings are outside of what might be expected at random. If the authors were to draw the same number of genes at random as found in each of the 3 classes (e.g. 1251 PAS genes) enough times to generate a reasonable null estimate, do their results of gene overlap and proportion fall into the tail of the null estimate?

4) Figure 1—figure supplement 4: There appears to be a strong bias in PAS usage favoring the chimpanzee samples. Can the authors explain this result? It isn't immediately obvious that we wouldn't expect a more normal distribution of PAS usage divergence between species.

Reviewer #3:

The manuscript by Mittleman and co-authors investigates alternative polyadenylation (APA) as a potential mechanism underlying the genetic regulation of transcript and protein expression levels in primates. Previous studies have identified genetic and epigenetic regulatory mechanisms underlying inter-species differences in gene expression. This is the first study focusing specifically on APA functional conservation/divergence between humans and chimpanzees. The manuscript describes APA in lymphoblastoid cell lines from six humans and six chimpanzees. The manuscript's main finding is that APA is largely conserved in humans and chimpanzees. Genes with significantly different PAS usage between the two species are enriched among differentially expressed genes, as well as among genes that show differences in protein translation between species. However, these results are based on relatively small subsets of genes. The manuscript is mainly focused on the molecular mechanisms and features of APA between species, while missing to investigate and discuss the biological role of the genes involved. This is problematic when trying to draw broader conclusions from analyses that focus on relatively small subset of genes, without any information on their biological relevance. For example, are the genes with differential APA and gene (or protein) expression relevant for divergent traits between the two species?

1) Differential PAS – The manuscripts reports 2,342 PAS, in 1,705 Genes, with differential usage between species. Additional information on the function of these genes should be reported as well as on the directionality of effect relative to the pathways and biological processes involved.

2) Signal site changes and PAS usage – The manuscript reports that the presence of a species-specific signal site is associated with increased PAS usage. Is this in the correct direction? In other words, does presence of the signal site in one species correspond to increased usage in the same species?

3) Differences in APA and gene expression – From the results in this section, it looks like the number of genes differentially expressed that also have differential PAS usage are a small subset. Is this because of lack of power (due to the small number of individuals included in the current study) or an actual biological phenomenon? How does this result compare to studies in humans of differential expression and differential APA in response to treatments or across cell types? What are the features and function of the genes represented in this subset?

4) The correlation in Figure 3B is only slightly higher than the one in Figure 3A, while the finding of opposite correlations depending on the location of the PAS used is interesting, these data do not support the interpretation. I would recommend moving this section to the supplements (supplemental figures) and keeping in the main text only the results of the analysis focused on genes differential expressed between species. Even in this case the trends aren't supported by strong results, so the test should be careful as to not overinterpret suggestive patterns. I would recommend to use Spearman's correlation rather than Pearson's because it is less sensitive to outliers. I would also suggest that the authors consider a scenario where the location of the PAS influences the direction of gene expression change, thus using a logistic model to test this hypothesis. Similar considerations apply also to the analysis of differential APA and differential protein expression.

5) Variation in APA and differences in protein expression – This section of the manuscript compares the APA data with published data of protein translation and expression in human and chimpanzee. While the enrichments and correlations reported in the first 2 paragraphs (and Figure 5) are of potential interest, the protein data are limited to a few thousand genes. How many of these genes can also be annotated in the APA data generated in this study? In other words, without the actual number of overlaps, it is difficult to assess whether the reported results are robust and widespread as opposed to limited to a few hundred genes. What are these genes functions? What can we learn about the evolution of human and chimpanzee-specific traits? Are these genes expected to show differential regulation between species? For the correlation analysis that considers intronic and 3'UTR PAS locations, please see my comment to the similar analysis done for differentially expressed genes. I recommend using Spearman's correlation and a logistic model.

# Reply: Author response

**DOI:** 10.7554/eLife.62548.sa2

We thank all of the reviewers for their thoughtful comments on our manuscript. All of the reviewers suggested that we include more biological information to support the mechanistic trends that we describe in the paper. In response, we performed a number of gene set enrichment analyses and added the results to the manuscript and supplement. We also provided additional information about the species-specific PAS example gene, and the genes previously annotated as subject to directional selection. The reviewers also asked us to perform functional validation of our RNA-seq data. In early RNA sequencing papers, authors performed validation experiments with qPCR. We now know that results from functional high throughput sequencing analyses can be trusted provided the appropriate quality control measures are used (as is the case for our study). The main aim of this work was to establish a genome-wide census of APA events in human and chimps and to infer global mechanisms that underlie species specific APA. Thus, our conclusions are generally based on aggregated observations from many sites and are robust against false positives and false negative errors. Like the reviewers, we acknowledge the importance of functional follow-up on our results to understand the effect of APA differences on phenotypes. However, the editors have agreed that follow-up experiments are outside the scope of this manuscript. We believe this study opens the door for using a similar study design to understand APA conservation in more cell types and dynamic biological processes. We added a few sentences to the discussion to acknowledge this limitation and reaffirm the goals of the study. Please see below for specific responses and changes we have made to the manuscript.

Reviewer #1:

The paper is a well-executed and thorough analysis of PAS usage in LCLs between humans and chimpanzees. The research is technically sound, and this is additionally demonstrated by the accompanying supplemental analyses, and the use of additional datasets which permitted studying how differential PAS usage relates to protein level expression. Yet the paper lacks direct connections to actual phenotypic and biological differences between humans and chimpanzees, and in this regard reads more of a technical work (perhaps suitable in its current form in a journal such as Genome Research or Nucleic Acids Research), than the type of study that is published on a specific biological phenotype as often presented in eLife. Along with additional issues listed below, are suggestions for adjusting the analyses and text to help bridge the gap to phenotype:

1) Throughout the Results sections the authors present a myriad of lists of gene and PAS usage sites that result from different ways of cutting the data and connecting PAS usage to isoform and protein expression. Can these lists be explored in more detail, perhaps through functional gene set enrichment analyses and/or the use of the GREAT? Analyzing the sets in at least this manner might help to connect PAS usage differences to actual biology between humans/chimpanzees as well as within each species.

We have now conducted a number of gene enrichment analyses using both fgsea and Gorilla (the details of which are found in the Materials and methods). We found that the differentially expressed genes within the dAPA genes are enriched for RNA processing pathways, such as RNA catabolic process and mRNA metabolic processes. We also found a 32X enrichment of translation initiation genes within the dAPA genes that are also differentially translated.

Using all of the dAPA genes as a background, we found functional enrichments for the genes that are differentially expressed in protein and not in mRNA. We identified small but significant enrichments for a number of sets related to vital cellular processes such as ribonucleotide binding, protein-containing complex binding, nuclear transport, and nucleocytoplasmic transport. We also found a number of gene regulatory components and processes enriched for genes with species-specific PAS compared to all genes where we identified a PAS.

We added these results to the Results and Materials and methods section of the paper. We also added two additional supplemental tables with the results.

2) While this reviewer greatly appreciated the assessment of PAS via 3' Seq along in tandem with mRNA expression, and in the context of their incorporation of published protein and isoform data on the same cell type, another main issue is the lack of any functional validation experiments for any of the insights they generate in each of the Results sections. This is a bit concerning considering the reliance on LCLs as a standalone cell type for this work.

It’s a balance. By using LCLs we were able to consider the APA data in the context of many other data sets collected from the same lines. Indeed, the greatest advantage of the LCLs is that they are a renewable resource that is available from multiple primate species. There is no other cell line that is available from multiple individuals from chimpanzees, other than our own panels of iPSCs. Certainly, we hope that future studies will consider other cell types based on this panel.

Reviewer #2:

Mittleman and colleagues have used 3' RNA-seq to study change in polyadenylation site usage between humans and chimpanzees. The results I suspect will be of greatest interest are that PAS change is associated with change in both RNA and protein level gene expression. This was a very interesting study, and something I hadn't considered before. The data collected seems to be a perfect fit for their study and the general analysis approach is solid.

1) I had one major concern with the manuscript as it's written-throughout the results, effect sizes were consistently weak to moderate at best (1-1.5X enrichment, weak correlations, etc.). This in itself is fine. I strongly believe there is often too much emphasis on results with large effect sizes. That said, the Discussion makes some strong claims about their data without tempering their language to account for the effect size. Some additional background information contrasting their results with other aspects of the transcription process would likely help place the role of PAS change in gene expression divergence between species. For example, how strongly does change in ChIP-seq/ATAC-seq/DNase-seq signal associate with DE genes between species. Basically, statements like "We showed that, across species, increased intronic PAS usage is associated with increased mRNA expression levels, while increased 3' UTR PAS usage is correlated with a decrease in mRNA expression" could use some additional context to highlight the relative significance of this result.

Thanks for the comment. We added the word modest to the sentence.

2) "Indeed, we found that inter-species differences in the usage of intronic and 3' UTR PAS correlate with differences in expression effect size between the species at an equal magnitude, but in opposite directions (Figure 3B). Increased usage of intronic sites is correlated with increased expression levels, while increased usage of 3' UTR sites is correlated with decreased expression."

It's unclear why the uncategorized correlation (-0.06) was deemed "not meaningful", yet the categorized correlations (-0.077, 0.073) were considered to be "correlated". Neither of these seem particularly well correlated to me.

We also agree that the correlations in Figure 3B are not strong. This is part of the reason we also present Figure 3D. When we subset on significant genes the correlation is stronger. We revised the language in this section to temper the results in 3B and demonstrate that the small correlation motivated our subsequent sub-setting of the data.

3) Figure 6 could use additional null models to determine whether these findings are outside of what might be expected at random. If the authors were to draw the same number of genes at random as found in each of the 3 classes (e.g. 1251 PAS genes) enough times to generate a reasonable null estimate, do their results of gene overlap and proportion fall into the tail of the null estimate?

We performed this analysis and found the actual number of differential APA genes that are differentially expressed in protein and not mRNA is not significantly higher than what would be expected by change (Author response image 1). The protein data comes from Khan et al. who measured 3,390 proteins with high resolution mass spec, thus analysis is fairly low powered. We are only able to sample 661 genes from 2632 genes that we have all of the data for. In addition, we do not expect APA differences to explain all or even a majority of the genes differentially expressed in protein but not mRNA. We expect a number of additional mechanisms to lead to protein differences.

**Author response image 1.**

4) Figure 1—figure supplement 4: There appears to be a strong bias in PAS usage favoring the chimpanzee samples. Can the authors explain this result? It isn't immediately obvious that we wouldn't expect a more normal distribution of PAS usage divergence between species.

We thank the reviewer for noticing this in the supplemental figure. We also found this unexpected at first, but we believe the result is an artifact of our QC and filtering.

PAS usage is a ratio of the reads mapping to one PAS over the number or reads mapping to any PAS assigned to the same gene. We calculated the usage values on an inclusive set of PAS then filtered to PAS reaching 5% in either species. We decided to identify and quantify sites this way to account for species and genomic region-specific noise. As a result of this choice, the overall usage for every gene may not add up to exactly 100%. This means that even though we identify (on average) the same number of PAS per gene in each species, the structure in this plot is not unexpected.

The bias toward chimpanzee in the supplemental figure shows that PAS usage is spread more evenly across human PAS than chimpanzee PAS. Before calculating PAS usage for PAS, we needed to assign sites to genes. Because the human annotation is more sophisticated than the chimpanzee annotation, we annotated all of the PAS to the human annotation. We acknowledge that if many PAS in chimpanzee fall outside of the human annotated genic regions, we would have lost those sites and the bias could result in the structure seen in the supplement. This would occur because included chimpanzee PAS would have inflated usage ratios. However, in reality we lost more of the sites originally discovered in human because they fell outside of the annotation than chimpanzee (22,278 in human vs. 18,954 in chimpanzee).

We also do not think the structure is a result of technical factors. We performed PCA on PAS usage (Figure 1—figure supplement 5). The top PC explains 41.8% of the variation and is highly correlated with species. The second PC explains 13.1% of the variation and is slighty correlated with extraction date and the author who collected the data. Because we balanced these technical factors with respect to species in the original study design (Supplementary file 4), the technical factors likely do not contribute to the structure the reviewer identified in Figure 1—figure supplement 4.

We account for any structure in the data introduced by the ratio characteristic of the phenotype in the differential PAS usage analysis. We normalized the usage values before testing for differences.

The PAS usage bias could also have affected the dominance analysis. However, anytime we refer to the dominant PAS in the manuscript, we show robustness of the results by presenting the results at a range of cutoffs.

Reviewer #3:

The manuscript by Mittleman and co-authors investigates alternative polyadenylation (APA) as a potential mechanism underlying the genetic regulation of transcript and protein expression levels in primates. Previous studies have identified genetic and epigenetic regulatory mechanisms underlying inter-species differences in gene expression. This is the first study focusing specifically on APA functional conservation/divergence between humans and chimpanzees. The manuscript describes APA in lymphoblastoid cell lines from six humans and six chimpanzees. The manuscript's main finding is that APA is largely conserved in humans and chimpanzees. Genes with significantly different PAS usage between the two species are enriched among differentially expressed genes, as well as among genes that show differences in protein translation between species. However, these results are based on relatively small subsets of genes. The manuscript is mainly focused on the molecular mechanisms and features of APA between species, while missing to investigate and discuss the biological role of the genes involved. This is problematic when trying to draw broader conclusions from analyses that focus on relatively small subset of genes, without any information on their biological relevance. For example, are the genes with differential APA and gene (or protein) expression relevant for divergent traits between the two species?

1) Differential PAS – The manuscripts reports 2,342 PAS, in 1,705 Genes, with differential usage between species. Additional information on the function of these genes should be reported as well as on the directionality of effect relative to the pathways and biological processes involved.

We added a number of gene set enrichments to the paper. Please see the general comments above.

2) Signal site changes and PAS usage – The manuscript reports that the presence of a species-specific signal site is associated with increased PAS usage. Is this in the correct direction? In other words, does presence of the signal site in one species correspond to increased usage in the same species?

Yes, this is the correct direction. Presence of a signal site in the species corresponds to increased usage of the site in the same species. This is best seen in our example figure, Figure 1—figure supplement 12.

3) Differences in APA and gene expression – From the results in this section, it looks like the number of genes differentially expressed that also have differential PAS usage are a small subset. Is this because of lack of power (due to the small number of individuals included in the current study) or an actual biological phenomenon? How does this result compare to studies in humans of differential expression and differential APA in response to treatments or across cell types? What are the features and function of the genes represented in this subset?

For the gene functions please see comments to other reviewers above.

4) The correlation in Figure 3B is only slightly higher than the one in Figure 3A, while the finding of opposite correlations depending on the location of the PAS used is interesting, these data do not support the interpretation. I would recommend moving this section to the supplements (supplemental figures) and keeping in the main text only the results of the analysis focused on genes differential expressed between species. Even in this case the trends aren't supported by strong results, so the test should be careful as to not overinterpret suggestive patterns. I would recommend to use Spearman's correlation rather than Pearson's because it is less sensitive to outliers. I would also suggest that the authors consider a scenario where the location of the PAS influences the direction of gene expression change, thus using a logistic model to test this hypothesis. Similar considerations apply also to the analysis of differential APA and differential protein expression.

We have added Spearman’s correlations to the legend in Figure 3. The results are consistent. We do not know how to perform a logistic regression because we do not know what continuous variable we would use.

5) Variation in APA and differences in protein expression – This section of the manuscript compares the APA data with published data of protein translation and expression in human and chimpanzee. While the enrichments and correlations reported in the first 2 paragraphs (and Figure 5) are of potential interest, the protein data are limited to a few thousand genes. How many of these genes can also be annotated in the APA data generated in this study? In other words, without the actual number of overlaps, it is difficult to assess whether the reported results are robust and widespread as opposed to limited to a few hundred genes. What are these genes functions? What can we learn about the evolution of human and chimpanzee-specific traits? Are these genes expected to show differential regulation between species? For the correlation analysis that considers intronic and 3'UTR PAS locations, please see my comment to the similar analysis done for differentially expressed genes. I recommend using Spearman's correlation and a logistic model.

For the gene functions please see comments to other reviewers above. We do not have a great way to annotate if a gene is expected to show differential regulation between species other than by overlapping the genes with other regulatory traits as we have done here. We note in the results the number of genes that we have APA and protein data for (3,391) and the number of genes we tested for expression and APA (7,462).
